# Clinical Characteristics, Intraoperative Findings, and Surgical Outcomes of Canalicular Laceration Repair with Monocanalicular Stent in Asia

**DOI:** 10.1155/2019/5872485

**Published:** 2019-07-02

**Authors:** Chun-Hsien Lin, Chun-Yuan Wang, Ying-Cheng Shen, Li-Chen Wei

**Affiliations:** Department of Ophthalmology, Taichung Veterans General Hospital, 1650 Taiwan Boulevard, Sec. 4, Taichung 40705, Taiwan

## Abstract

**Purpose:**

To report the epidemiological and clinical data as well as surgical outcomes of canalicular lacerations with Mini-Monoka insertion at a tertiary center in Taiwan and to discuss differences in traumatic pattern, pathogenesis, and surgical outcomes between Taiwan and other countries.

**Methods:**

From 2009 to 2018, all 48 patients who underwent canalicular laceration repair with Mini-Monoka stent at a tertiary center in Taiwan were retrospectively analyzed. Demographic and clinical data and surgical outcomes were recorded.

**Results:**

The mean age of the 48 patients was 38 years. Single lower canaliculus was involved in 37 (77.1%) patients, upper canaliculus in 10 (20.8%) patients, and both in 1 (2.1%) patient. The most common etiology was motorcycle accident (41.7%), and all traffic accident injuries accounted for 68.75% of cases. Subgroup classification revealed 64.6% of patients (*n*=31) were categorized in the deep laceration group, and lower anatomical and functional outcomes were noted in deep laceration. The mean follow-up time was 14.5 months. Overall, the anatomical success rate was 87.5%, and the functional success rate was 91.7% after stent removal.

**Conclusion:**

Canalicular laceration caused by traffic accidents occurred with a relatively high frequency in Taiwan. Affected patients tended to be middle-aged, and deep laceration accounted for 64.6% of patients. These were contributed by the avulsive eyelid injury mechanism caused by traffic accidents. Furthermore, the deeper lacerated site was located, and the lower anatomical and functional success rates were observed. Early repair after trauma with Mini-Monoka stents achieved good eyelid position (100%) as well as good anatomical (87.5%) and functional (91.7%) success without serious complication.

## 1. Introduction

Eyelid injuries are sometimes accompanied by canalicular laceration because the canaliculus sits just beneath the thin layer of the eyelid skin without additional protection [1]. The lower canaliculus is especially vulnerable to direct penetrating and indirect or diffuse avulsive blunt injury to pericanalicular soft tissue in the lacrimal drainage system [1, 2]. According to the previous studies, 16–36% lesions of the lacrimal drainage system were noted in all eyelid injuries [3–5]. If not properly managed, a number of sequelae such as ectropion, epiphora, and poor cosmetic appearance could occur.

Most ophthalmologists recommend immediate management with stenting of the lacerated canaliculus to successfully restore proper eyelid anatomy, prevent medial ectropion, and prevent canalicular obstruction [6, 7]. If the patient delays seeking medical attention or the surgery is delayed, visualization of the medial end of the lacerated canaliculus is difficult and fibrosis or epithelialization at the cut ends may be present [8]. Lacrimal drainage system blockage will result in epiphora due to canalicular stenosis, pericanalicular scarring band, or malposition of the punctum [1].

There are several surgical techniques for management of canalicular laceration [9–12]. The current consensus is surgical placement of canalicular stent or intubation. Historically, a single-lacerated canaliculus could be managed with a pigtail probe with annular stent or bicanalicular nasal intubation [6, 13]. The pigtail probe method was less preferred in the recent years due to high risk of injuring the nonlacerated canaliculus, modest functional and anatomical outcome, and the high level of surgical expertise required despite the development of round-tipped pigtail probe [14–18]. Bicanalicular nasal intubation can be used in monocanalicular and bicanalicular lacerations. However, there are some reported complications about nasal intubation, including false passage, punctal or canalicular slitting, granuloma formation, superior loop dislocation, corneal abrasion, and inadvertent trauma to the uninvolved canaliculus [19–22].

**Figure 1:**
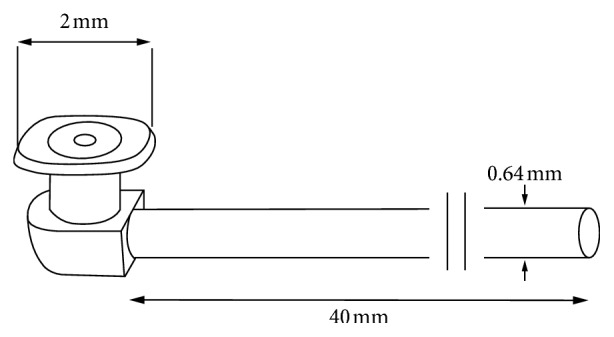
The Mini-Monoka stent is securely anchored at the punctum by the 2 mm collarette. No knots or sutures are necessary.

Various materials, such as polyethylene, metal rod, and silicone, have been used in producing the lacrimal stent. In recent years, medical-grade silicone has become the mainstay material for lacrimal stent because of its inert chemical properties and flexibility [3, 6, 8, 23, 24]. The Mini-Monoka monocanalicular stent, which is made of silicone and coated with polyvinylpyrrolidone, is widely used for congenital or acquired nasolacrimal duct obstruction and canalicular laceration [25]. The diameter of the silicone tube is 0.64 mm, and it has a 2 mm collarette at the proximal end (Figure 1), which securely anchors the stent at the punctum obviating the need for knots or sutures.

The purpose of this study was to report the epidemiological data, clinical profile, and surgical outcomes of canalicular lacerations with Mini-Monoka insertion at a tertiary center in Taiwan and to discuss differences in traumatic pattern, pathogenesis, and surgical outcomes between Taiwan and other countries.

## 2. Materials and Methods

In this retrospective case series, records of all patients who underwent canalicular laceration repair with Mini-Monoka monocanalicular stent from January 1, 2009, to April 30, 2018, at a tertiary center in Taiwan were collected and analyzed. The study protocol was approved by the Institutional Review Board, and the tenets of Declaration of Helsinki were followed. Patients whose follow-up duration was shorter than 6 months were excluded. The data collected included demographics, cause of eyelid injury, distance between lateral canalicular lacerated end and punctum (which was divided into 3 subgroups: <4 mm, 4–7 mm, and ≥7 mm), duration from injury to surgery, associated ocular injury, surgical outcomes, time of stent removal, and follow-up duration. Anatomical success was defined as a patent lacrimal sac irrigation and patent canaliculus on diagnostic probing (hard stop). Functional success was defined as absence of epiphora after stent removal.

The most challenging aspect of the surgery is to identify the medial cut end of lacerated canaliculus. In addition to direct inspection of the pinkish tubular canalicular mucosal tissue and traction surrounding soft tissue, we also used saline or diluted povidone-iodine injection through the opposite punctum while maintaining pressure over the lacrimal sac. After injection, the flow of saline or povidone-iodine from the medial cut area facilitated identification of the cut end of the canaliculus [22, 26]. After identifying the medial cut end, a number-0 or number-1 Bowman lacrimal probe was used to pass through both cut ends and reach the lacrimal sac with hard stop. Then the punctum was dilated. Finally, Mini-Monoka stent was inserted with its distal end passing into the lacrimal sac or nasolacrimal duct, and the proximal end was fixed securely over the punctum with its collarette. Two to three pericanalicular sutures with 5-0 polyglactin were placed to fix and maintain the lacerated canaliculus in a proper anastomosis, and associated eyelid laceration was repaired with 6-0 silk suture. All of the surgeries were done by the chief resident or a fellow. The Mini-Monoka stent was planned to be removed 5-6 months after operation.

## 3. Results

**Figure 2:**
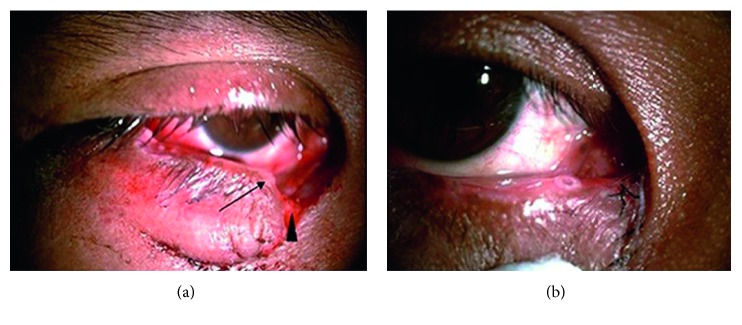
Right lower canalicular laceration in a 36-year-old male patient was sustained in a bicycle accident. (a) In a preoperative photograph, an arrowhead shows the lateral cut end of the lower canaliculus and an arrow shows the punctum. (b) One week after trauma, the Mini-Monoka monocanalicular stent was placed and the eyelid was in a good position without ectropion.

A total of 48 patients with canalicular laceration underwent stenting with the Mini-Monoka stent during the 9-year period of investigation (Figure 2). The mean age at presentation was 38 years (range, 3 to 73 years), 33 (68.75%) patients were male, and 15 (31.25%) were female. The most common etiology was motorcycle accident (20 patients, 41.7%). Other causes of injuries included bicycle accident in 10 (20.8%), car accident in 3 (6.3%), fight injury in 5 (10.4%) patients, work-related injury in 5 (10.4%), fall-related trauma in 3 (6.3%), and dog bite in 2 (4.2%) patients. Canalicular disruptions in the left eye occurred in 29 (60.4%) patients and occurred in the right eye in 19 (39.6%) patients. The lower canaliculus was involved in 37 (77.1%), upper one in 10 (20.8%), and both in 1 (2.1%) patient. Simultaneous globe injury was noted in 5 (10.4%) patients, and orbital wall fracture was found in 3 patients (6.25%). The demographic data of the patients are summarized in Table 1.

We classified our patients into 3 subgroups on the basis of distance from the lateral end of canalicular laceration to the punctum. There were no patients in the shallow laceration group, which was defined as a distance from the lacerated end to the punctum measuring less than 4 mm. There were 17 patients in the moderate laceration group, which was defined as a distance from the lacerated end to the punctum measuring 4–7 mm. There were 31 patients in the deep laceration group, which was defined as a distance from the lacerated end to the punctum measuring greater than 7 mm. Most of our cases were categorized in the deep laceration group (64.6%, 31 of 48 patients), and in over 80% of patients (*n*=40), the distance between laceration and punctum was greater than 6 mm, which made surgery more difficult. The details of distance from the cut ends to the punctum in each case are summarized in Table 2.

The stent was left in situ from 3 to 25 weeks (mean, 20.2 weeks). Spontaneous premature extrusion of stent (<1 month) was noted in 3 patients at 3, 4, and 4 weeks postoperatively, and anatomical failure was noted in 2 of these 3 patients on diagnostic probing or irrigation after stent extrusion. The mean duration of follow-up in all patients was 14.5 months.

After removing the Mini-Monoka stent, good eyelid position was achieved in all patients (100%) without ectropion or entropion. At the final follow-up (mean, 14.5 months), the anatomical success rate was 87.5% (42 patients), which means canalicular block was noted during diagnostic probing or lacrimal irrigation in 6 (12.5%) (including 2 premature extrusion cases) out of 48 patients. The functional success rate was 91.7% (44 patients) with 4 (8.3%) patients exhibiting persistent epiphora. Subgroup analysis revealed that both anatomical success rate and functional success rate were 94.1% in the moderate laceration group, whereas the anatomical and functional success rates in the deep laceration group were 83.9% and 90.3%, respectively. The only patient with both upper and lower canalicular lacerations had patent canaliculus with no epiphora at the last follow-up (15 months after surgery). There were no other postoperative complications, except premature extrusion of stent. The surgical outcomes of the patients are summarized in Table 2.

## 4. Discussion

The Mini-Monoka monocanalicular stent and bicanalicular nasal intubation are the mainstay of canalicular laceration repair in recent decades. However, repair with bicanalicular intubation does have some drawbacks. Nasal intubation requires a special hook or endoscopic guidance to retrieve the tube from nasal cavity, and surgeons must perform probing precisely to the lower lacrimal system; otherwise, there is a risk of creating a false passage, which would hinder successful intubation [6, 13]. In contrast, Mini-Monoka insertion does not require retrieval of a tube from nasal cavity and it is not necessary to perform probing to the lower lacrimal system. Hence, compared with the Mini-Monoka stent, bicanalicular intubation is more time-consuming and demands more surgical expertise and experience. In our series, all of the surgeries were done by the chief resident or a fellow. They have less experience and have comparatively less advanced surgical skills compared with oculoplastic specialist, so most cases of canalicular laceration are managed with the Mini-Monoka stent in our hospital. Moreover, there was one patient with both upper and lower canalicular lacerations in our series. We also performed two Mini-Monoka stents rather than a bicanalicular stent, as described in previous studies [3, 24, 27, 28]. After removing the two Mini-Monoka stents, both upper and lower canaliculi demonstrated patency without epiphora at 15 months after surgery.

According to the previous studies, canalicular laceration frequently occurred in children and young adults. Kennedy and associates, in an 11-year clinical study, noted that 68% of canalicular injuries occurred in persons below 30 years of age [2]. Naik and associates conducted a clinical study in India on canalicular laceration, and the mean age of patients was 16 years old [3]. In 2017, Alam and associates in a similar study in India found the mean age of patients was 19.3 years old [8]. However, in our study, the mean age of patients with canalicular laceration was 38 years old with only 10.4% below 20 years of age. We postulate that the large difference in mean age between our study and that of other series was due to the variation in injury mechanism. In the two Indian studies, the most common cause of canalicular laceration was penetrating injury by blouse-hook fastener in infants while breastfeeding [2]. In Kennedy's study, although the most common cause of eyelid injury was blows from fists, it only accounted for 23.4% of all injured cases. Dog bites or scratches, which involve a penetrating mechanism, accounted for a certain proportion of all cases and were the most frequent etiology among children [2].

In our series, the most common etiology was the motorcycle accident. According to the Ministry of Transportation and Communication of Taiwan, in 2018 (https://stat.motc.gov.tw/mocdb/stmain.jsp?sys=100&funid=a3301), there were 13.8 million motorcycles, i.e., 2 motorcycles for every 3 persons in Taiwan and 24 thousand traffic accidents per month. The uniquely high per capita motorcycle ownership and high rate of traffic accidents in Taiwan may account for the main etiology of eyelid avulsive injury and concomitant canalicular laceration in our patients and may explain why our patients tended to be middle-aged rather than children. Moreover, in contrast to the two aforementioned Indian studies in which most of the injury mechanisms involved penetrating injury, in our series, over 60% of patients suffered from blunt or avulsive eyelid injury due to traffic accident. It might also explain the difference in simultaneous globe injury between our series (10.4%) and previous reports (20–44%) [3–5, 8]. A male predominance (75%) was noted in our series, which also could be explained by the higher motorcycle usage rate in males.

In the present series, we measured the distance from the lateral lacerated ends to the punctum and classified patients into shallow, moderate, and deep laceration groups. Most of our cases had a laceration measuring greater than 7 mm (31 out of 48, 64.6%), which were categorized in the deep laceration group. We postulate the reason that generally large laceration distances in our cases may have been due to the large percentage (68.8%) of blunt eyelid injury mechanism caused by traffic accident. In contrast to the two abovementioned Indian studies, which showed that the penetrating injury was the main injury mechanism, we speculate that blunt eyelid injury secondary to lateral shearing forces may cause deeper canalicular laceration, because the lateral shearing force passes the eyelid's elastic limit and ruptures at the weak point, the deeper canaliculus nearby the medial canthal tendon.

Compared with the deep laceration group, the moderate laceration group in our series revealed better anatomical and functional outcomes. It is intuitive that the deeper laceration made surgery more difficult as it was hard to identify the medial cut end and the higher wound tension to place the suture properly. Thus, we postulate that worse canalicular anastomosis might be expected in deeper laceration and resulted in lower anatomical and functional success rate. Singh and associates also reported better anatomical outcome in proximal laceration in their series. Otherwise, in the Fluorescein Dye Disappearance Test, the positive rate, which meant dye still persisted 5 minutes after instillation of fluorescein drop, was higher in the proximal laceration group. They speculate the functional failure in the proximal laceration group was secondary to compromised lacrimal pumping due to injury of Horner muscle and orbicularis oculi [29]. However, in our study, 94.1% patients did not complain of epiphora in the moderate laceration group. Even pumping function of the lacrimal system might be impaired after canalicular laceration, and most patients did not suffer from the symptom of epiphora if the residual function and opposite canalicular function were sufficient to excrete the tear.

According to the previous studies, after canalicular laceration repair, the rate of premature extrusion of the Mini-Monoka stent varied. Anastas and associates reported 29% of their cases experienced premature stent loss following treatment with the Mini-Monoka stent [24]. Naik and associates reported premature stent extrusion or migration occurred in 11.1% of the 27 patients analyzed in their series [3]. Similarly, Kim and associates reported 7.8% of patients encountered premature stent loss in their series [30]. In our study, premature extrusion was noted in 6.25% of patients (3 of 48 patients). The first extrusion happened in a 3-year-old girl; total extrusion occurred at 3 weeks after trauma due to self-removal of the stent. Diagnostic probing and lacrimal irrigation both revealed blocked canaliculus. The second case of extrusion in a 7-year-old boy with partial stent extrusion was due to the same reason at 4 weeks postoperatively. Mini-Monoka repositioning was done, and anatomical and functional success was noted after the elective stent removal. The third case of stent extrusion occurred in a 52-year-old man at the fourth week postoperatively. We postulate that it might have been related to excessive punctal dilation during surgery. Diagnostic probing and lacrimal irrigation also showed blocked canaliculus. Finally, the patient received conjunctivodacryocystorhinostomy 3 months after trauma with no subsequent complaint of epiphora thereafter. In our cases, premature extrusion seemed to predispose patients to a higher chance of anatomical block (2 out of 3 patients). There are two possible reasons for this phenomenon. First, premature stent extrusion leads to disruption of pericanalicular tissue healing. It may cause peri- or intracanalicular fibrosis and stenosis or canalicular anastomosis misalignment. Second, cut-end distances from the punctum of our 3 extrusion cases were large (7, 9, and 10 mm, respectively). It is more difficult to place sutures in a deeper plane, and thus tissue apposition in such cases might not be as satisfactory as in cases with a shallower laceration, which would tend to result in a higher anatomical failure rate. Most of our patients could tolerate and care the tube well until the tube removal at 5-6 months after trauma. The lower extrusion rate in our series might be due to the relatively small number of pediatric cases compared with previous series in other countries, which meant fewer events of stent self-removal.

The previous studies revealed a functional success rate of 94–100% with the Mini-Monoka stent [1–3, 31]. In our series, the anatomical success rate was 87.5% (42 out of 48 patients), and the functional success rate was 91.7% (44 out of 48 patients). There are some possible reasons our success rate was lower than in the previous studies as follows. First, all of our operations were done by the chief resident or a fellow. Their surgical experience and level of expertise may not have been on par with a senior oculoplastic specialist. Second, the etiology in most of our cases was traffic accident with avulsive eyelid injury and the distance from the lacerated end to the punctum was generally long. Singh and associates reported that the factors predictive of poor outcome were related to the mode of injury, especially road traffic accidents and skill of the surgeon [32]. Murchison and associates also reported that level of surgical training and performing the repair in a minor procedure room, rather than in the operating room, will decrease the success rate [33]. Our study findings are consistent with the results of these two studies. Alam and associates reported an anatomical success rate of 85.7% and a functional success rate of 92.85% in their series [8]. Their lower success rate might be attributed to the fact that 10 out of 29 patients presented more than 11 days after trauma, which made surgery more difficult. There is general agreement on the need for immediate stenting of canalicular injuries, because late repair frequently indicates a difficult surgery and poor results [34]. However, some authors recently suggested that canalicular repair can be delayed for up to 11 days, and Chu also reported that, for experienced oculoplastic specialists, there was no difference between early (within 48 hours) and late repair (after 48 hours) in success rate and operation time [23, 35]. Nevertheless, we still recommend early stenting of canalicular injuries as it is easier to identify the medial lacerated end during operation. Hence, time from injury to surgery, as a possible confounding factor of surgical success, was not a problem in our study because all of the canalicular laceration repairs with Mini-Monoka stent in our study were done within 48 hours. For patients with delayed presentation and the canaliculus being the obstruction with a symptom of epiphora, an oculoplastic specialist will perform conjunctivodacryocystorhinostomy.

In this study, there were some limitations, such as the retrospective nature of the investigation and the lack of direct comparison of outcomes and complications between the Mini-Monoka stent and other surgical techniques.

## 5. Conclusions

In conclusion, in the majority of our cases, canalicular laceration was caused by a unique injury mechanism, due to the remarkably high per capita motorcycle ownership and high rate of traffic accidents in Taiwan. The patients were largely middle-aged, and the lacerated sites were generally deep. These were contributed by the avulsive eyelid injury mechanism caused by traffic accidents. Furthermore, the deeper the lacerated site is located, the lower anatomical and functional success rates were observed. Although the surgeries in our case series were performed by surgeons with less experience (i.e., the chief resident or a fellow), early repair after trauma with Mini-Monoka stents achieved good eyelid position (100%), good anatomical (87.5%) success, and good functional (91.7%) success without serious complication.

## Figures and Tables

**Table 1:** Demographic data of patients with lacerated canaliculus repaired with the Mini-Monoka stent.

Characteristics	Number (%)
Total no. of patients	48 (49 canaliculi)
Mean age	38 (3–73 years)
Male	33 (68.75)
Female	15 (31.25)
Canaliculus involvement	
Upper	10 (20.8)
Lower	37 (77.1)
Both	1 (2.1)
Etiology of injury	
Motorcycle accident	20 (41.7)
Bicycle accident	10 (20.8)
Car accident	3 (6.3)
Fight injury	5 (10.4)
Work-related injury	5 (10.4)
Fall-related trauma	3 (6.3)
Dog bite	2 (4.2)
Mean duration from injury to surgery	10 hours

**Table 2:** Surgical outcomes following placement of 49 Mini-Monoka monocanalicular stents in 48 patients with canalicular lacerations.

Feature	Number (% or range)
Mean duration of stent	20.2 weeks (3–25 weeks)
Mean follow-up	14.5 months (6–18.5 months)
Distance from laceration to punctum	
Shallow laceration group: <4 mm	*n*=0 (0%)
Moderate laceration group: 4–7 mm	*n*=17 (35.4%); 4-5 mm, *n*=3; 5-6 mm, *n*=5; 6-7 mm *n*=9
Deep laceration group: ≥7 mm	*n*=31 (64.6%); 7-8 mm, *n*=11; 8-9 mm, *n*=11; 9-10 mm, *n*=9
Premature extrusion, *n* (%)	3 (6.25)
Postoperative canalicular block, *n* (%)	6 (12.5)
Anatomical success, *n* (%)	
Overall (*n*=48)	42 (87.5)
Moderate laceration group (*n*=17)	16 (94.1)
Deep laceration group (*n*=31)	26 (83.9)
Functional success, *n* (%)	
Overall (*n*=48)	44 (91.7)
Moderate laceration group (*n*=17)	16 (94.1)
Deep laceration group (*n*=31)	28 (90.3)

## Data Availability

The data used to support the findings of this study are included within the article.
